# Dog growls express various contextual and affective content for human listeners

**DOI:** 10.1098/rsos.170134

**Published:** 2017-05-17

**Authors:** T. Faragó, N. Takács, Á. Miklósi, P. Pongrácz

**Affiliations:** 1 Department of Ethology, Biology Institute, Eötvös Loránd University, Pázmány Péter stny. 1/C, Budapest, H-1117Hungary; 2 MTA-ELTE Comparative Ethology Research Group, Pázmány Péter stny. 1/C, Budapest, H-1117Hungary

**Keywords:** emotion recognition, dog–human communication, growl, bioacoustics, vocal expression of emotions

## Abstract

Vocal expressions of emotions follow simple rules to encode the inner state of the caller into acoustic parameters, not just within species, but also in cross-species communication. Humans use these structural rules to attribute emotions to dog vocalizations, especially to barks, which match with their contexts. In contrast, humans were found to be unable to differentiate between playful and threatening growls, probably because single growls' aggression level was assessed based on acoustic size cues. To resolve this contradiction, we played back natural growl bouts from three social contexts (food guarding, threatening and playing) to humans, who had to rate the emotional load and guess the context of the playbacks. Listeners attributed emotions to growls according to their social contexts. Within threatening and playful contexts, bouts with shorter, slower pulsing growls and showing smaller apparent body size were rated to be less aggressive and fearful, but more playful and happy. Participants associated the correct contexts with the growls above chance. Moreover, women and participants experienced with dogs scored higher in this task. Our results indicate that dogs may communicate honestly their size and inner state in a serious contest situation, while manipulatively in more uncertain defensive and playful contexts.

## 1. Introduction

During social interactions, both humans and non-human animals use various communicative signals to express their inner states. The way in which emotions are reflected in the acoustic structure of calls is best described by the Source-Filter Framework (for detailed review see [1]). In short, the specific changes in the brain due to emotional states can affect the neural control over the muscle movements involved in voice production in the larynx and the vocal tract, and these changes modify certain acoustic parameters of the produced calls [2]. On the one hand, these parameters can be source-related when the respiration or the phonation system is affected, causing changes in the amplitude, the call duration and the fundamental frequency. On the other hand, they can be filter-related due to the modification of the length or shape of the vocal tract affecting the spectral energy distribution in the sound, creating, for example, formant frequencies. Of these prominent frequency band position and distribution across the spectrum depends mainly on the length of the vocal tract, thus the so-called formant dispersion acts like an important indexical cue in communication [3].

A growing body of evidence suggests that in humans, specific brain regions are involved in processing these emotion-expressing vocalizations that are different from those that are responsible for speech perception [4,5]. Belin *et al*. found that the same brain centres are responsible for the processing of animal (cat and rhesus macaque) and human non-verbal vocalizations with a negative valence [6]. Moreover, our recent fMRI study showed that in dogs and humans, similar brain regions are involved in processing the emotional load of non-verbal vocal expressions [7], suggesting that the neurological processes of extracting emotional information from the acoustic structure of calls is shared among mammals.

Based on this we can assume that acoustic emotion recognition can work not only within species, but also in interspecific communication. Indeed, numerous studies have found several examples of adequate reactions to heterospecific alarm calls (e.g. ground squirrels [8]; mongooses [9]; sifaka and lemur [10]) or distress vocalizations [11]. Humans are also able to use these acoustic features to assess the inner state and decipher the contexts of non-human vocalizations (calls of macaques: [12]; pigs: [13,14]; dogs: [15]). Some results suggest that this recognition is affected by the experience level of the individual with the vocalizing species (e.g. domestic cats: [16,17], pigs: [18]). Scheumann *et al*. compared the emotion assessment performance of humans on human infant, chimpanzee, dog and tree shrew affiliative and aggressive calls, and found that familiarity with the given species strongly affected the context recognition success [19]. By contrast, in studies on dog barks, humans recognized most of the contexts above chance and their performance was only minimally affected by their experiences [15,20,21]. Furthermore, in the case of a wide range of dog and human non-verbal vocalizations, human participants used the same basic acoustic rules to assess the emotional load of the calls independently from the caller's species: shorter calls were associated with positive valence, while higher-pitched calls were rated as emotionally more intense [22].

Dog vocalizations are especially interesting when studying cross-species emotional communication, because of the long-shared history of the two species [23] and the possible effects of human selection on the vocal repertoire of dogs [24]. While barks seem to have become diversified contextually and acoustically over the course of domestication, growls seem to be less affected, as they are mostly used in similar contexts in dogs and their closest wild relatives [25]. Growls are effective in close distance communication and are emitted in both agonistic and playful contexts [26]. While the intraspecific communicative role of dog growls has already been tested (context: [27]; body size: [28–30]), our knowledge about the possible information of growls in dog–human communication is still sparse compared with that of barks. Taylor *et al.* [31,32] found that both fundamental frequency (*f*_0_) and formant dispersion (d*F*) convey size and inner-state information for humans: dogs emitting growls with lower *f*_0_ and closer formats were rated as being larger and more aggressive. Interestingly, it was also found that humans were unable to differentiate between the context of single, isolated growls originating from playful and aggressive situations [33]. Additionally, they found that using resynthesized bouts of growls with inter-growl intervals (IGI) manipulated to reflect typical growling rates in play or aggression contexts instead of single isolated growls, improved the listeners' recognition success. However, the length of the individual growls in the sequence was restricted to 1.2 s, which is shorter than average aggressive and longer than average playful growls. These results suggest that the natural temporal structure of these vocalizations play an important role in encoding the emotional state of the dogs.

Based on the above-mentioned results with growls and barks [15,34], we hypothesize that besides the source- and filter-related parameters, temporal patterns (call length, pulse) of repetitive vocalizations play a crucial role in encoding affective and contextual content. Thus, we aimed to explore whether humans recognize the context of natural (with unmodified frequency and time structure) growl sequences, and how they rate the inner state of dogs based on their growls. We used three contexts, a playful and threatening context (used by Taylor *et al.* [33]), and an additional food-guarding context. We asked the participants to rate the inner state of the dogs, not just on the playfulness and aggressiveness scales, but also on three other emotional scales (fear, despair and happiness), that had been previously used to rate dog barks (see [15]).

We assume that within the three types of growls (food guarding, threatening and play) the IGI and growl length will affect the ratings of positive valence, namely shorter and fast pulsing growls will be rated as more playful and happy [22], while lower pitch and formant dispersion will be associated with higher aggression, lower fearfulness and despair [33]. We hypothesize that using these features, humans can attribute inner states to these natural growl sequences (in contrast with single growls) and they are able to recognize correctly their contexts. Moreover, based on the higher sensitivity of women to emotional stimuli (e.g. [35]) and earlier ambiguous findings on the possible influence of experience on recognition success, the effect of participants' gender and dog experience was also examined.

## 2. Material and methods

### 2.1. Participants

Forty adult humans participated in our experiment (14 males and 26 females, age: 26.1 ± 7.4 years, for further details see electronic supplementary material, table S1). Participation was voluntary, and the subjects were informed that their data would be stored anonymously and handled confidentially. They had no prior information about the specific goals of the study. Participants were tested one by one, in the presence of an experimenter (N.T. or T.F.).

### 2.2. Stimuli

For the playbacks, we used dog growl recordings from the pool of vocalization sequences collected for an earlier acoustical analysis and playback study [27]. Three contexts were represented in the playback: a dog guarding food from a conspecific (food guarding), when threatened by a stranger (threatening) and when playing tug-of-war with the owner (play). For the exact description of the sound recording process, see [27]. We used 10 s sections from the original recordings that contained at least three growls with low background noise for the playbacks. We had eight different growl samples in each context recorded from 18 different dogs (eight males, 10 females; age: 4.18 ± 2.26; of various breeds and mongrels; electronic supplementary material, table S2). From the 24 available growl samples, we generated 20 different playback sets, each consisting of six samples, two from each context. Each growl sample was used in five sets, and within one set the growls from the same context originated from different dogs. In the playback sets, we avoided using two consecutive growls from the same context (electronic supplementary material, table S3). Each playback set was used for two listeners only, thus each individual growl sample was evaluated by 10 participants. At the end of the playback sets, we repeated the first growl of that sequence in order to measure the reliability of the responses.

### 2.3. Acoustic measurements

Using a custom-made Praat script [36] we measured the length (CL), *f*_0_ and formant dispersion (d*F*) of each individual growl within one recording, and also the time between offset and onset of each consecutive growl to get the IGI. Each parameter was averaged through each 10 s long sample and this average was used for characterizing the growl samples in the further analysis (electronic supplementary material, tables S2 and S4).

### 2.4. Questionnaires

Before the actual playbacks, the participants completed a questionnaire with basic background information on age, experience with dogs, and whether they have ever been bitten by a dog. During the playbacks, we used two scoring sheets, one for emotional scaling and one for context recognition. The participants completed the emotional scoring sheet first because we tried to avoid the impression that there are only three possible contexts (which could bias the scoring of the emotional content).

For the emotional scaling, the participants had to rate the growls by five inner states: aggression, fear, despair, happiness and playfulness. However, in contrast with the earlier studies, here we used a visual analogue scale (VAS) instead of a five-grade Likert scale. Participants had to place a mark on a 100 mm long horizontal line. The distance of the mark from the left end of the line in millimetres represented how much the participant felt that the given inner state was characteristic to the actual growl. This way the participants had the opportunity to choose from a wider scale, and this method also provides a finer discrimination of the growls, resulting in a continuous variable for our measurements [37]. The participants next had to choose one of the three possible contexts (Food guarding, Threatening, Play), using the context scoring sheet.

### 2.5. Procedure

The tests were performed in a quiet office room at the Department of Ethology, Budapest. During the playback session only the participant and one of the experimenters (who operated the playback) were present in the room. After a short explanation of the task, the participant sat at a desk with no visual access to the playback computer and was given the first sheet in which they had to scale the emotional content of the first playback set. After the experimenter explained the procedure and ensured that the participant understood the task, the growl samples were played from a PC using Adobe Audition 1.5, through a high-quality speaker system (Genius sw-5.1 Home Theatre). The playback was a sequence of growl samples with 30 s long pause each sample, thus the participants could easily scale the growl bouts one by one. After the end of the first set, we gave the participant the second sheet for categorizing the context of the growls in a second playback set. We played this second sequence in a similar way as the first one, but a different set was always used, with no overlapping growl samples between the two. If the participant requested it, we played particular growl samples once more.

### 2.6. Data analysis

Emotional scaling data were recorded in millimetres, growl by growl, for each inner state. We excluded from the main analysis the seventh, repeated growl playback, which was only used for the analysis of consistency. As we wanted to test the possible context-dependency of the ratings of inner states, we averaged the scores of each two growls from the same context rated by the same participant. Finally, we calculated the average scaling of each inner state given by the listeners, growl by growl, for the regression and correlation analysis.

In the case of contexts, participants' responses were compared with the original recording context of the growls. The correct assignment of the context was recorded as a binomial variable (correct–incorrect). For the confusion matrix, the percentage of the three given answers for all three growl types was calculated.

### 2.7. Statistics

To analyse how the context of growls affected the emotional ratings, we used generalized linear mixed models (GLMMs). As the averaged ratings were left skewed, we applied a Box--Cox transformation (power: 0.4). Besides the main factors (context and emotional scale), we included into the model the participants' demographic variables (categorical: gender, ownership, bite history; continuous: age). We applied *p*-value-based backward elimination approach to simplify our model, thus initially we included all variables and their two-way interactions, then the interactions and factors with the highest *p*-value were removed step-by-step. The simplest, final model is reported in the results.

The acoustic parameters were compared between the three contexts also with GLMMs, adding the dog ID as a random factor. For the comparison of the number of individual growls, we used models with Poisson distribution, while in the other cases Gaussian distribution with log link was used, as the data were left skewed.

For testing how the acoustic parameters of the growls affect the emotional ratings, we used separate linear regression models with backward elimination for each scale. As growls recorded from different contexts show marked acoustic differences [27], this analysis was performed in all three contexts separately.

In the case of context classification, binomial tests were used to compare the participants' performance to the random 33% correct classification. To test the effect of the demographic variables on the success rate, we applied mixed effects binary logistic regression model and backward elimination. In both models (emotion rating and context recognition), we used sequential Sidak post hoc tests for pairwise comparisons. In the results, the corrected *p*-values are reported.

Finally, the consistency of the emotional scaling and the context recognitions were analysed with Spearman correlation tests (see electronic supplementary material, results and figure S1).

All statistics were performed in SPSS 22, figures were generated in R with ggplot2 package.

## 3. Results

### 3.1. Assessment of emotions in the growls

**Figure 1.**
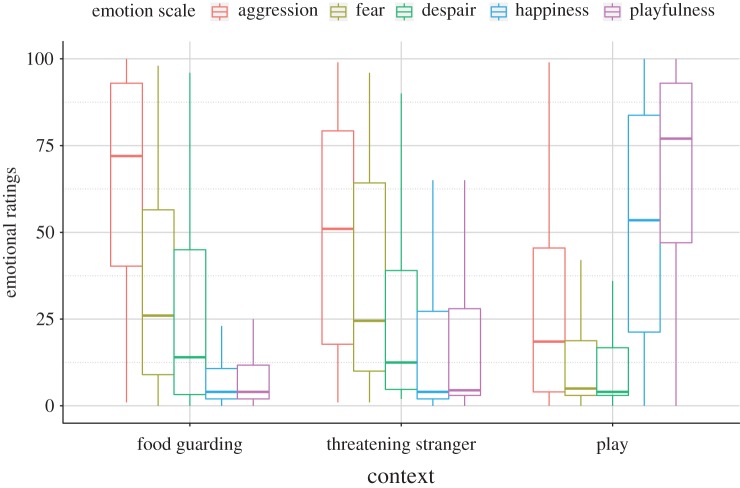
Medians of the emotional ratings between the three growl types. The boxes indicate the interquartiles, the whiskers the lowest and highest non-outlier values.

The final GLMM on the emotional ratings (overall lr test: *F*(14;585) = 29.862; *p* < 0.001) showed a significant interaction between the context of the growls and the emotion scales (interaction effect: *F*(8;585) = 40.366; *p* < 0.001) suggesting that the participants rated differently the emotional background of the growls from the different contexts (figure 1). None of the demographic variables, nor their interactions, had a significant effect on the emotional ratings, and thus were not included in the final model.

The post hoc tests revealed that the context had a significant effect on the assessment of emotions. All three contexts differed in scores of aggression: playful growls were rated the lowest, and the two agonistic contexts also differed with the food-guarding growls scoring the highest on the aggression scale. Regarding the other four emotional scales, the two agonistic growl types did not differ significantly, while the playful growls were rated significantly higher on playfulness and happiness and lower on despair and fearfulness (*p-*values in electronic supplementary material, table S5).

Food-guarding growls were scored highest on aggression, and lower on fear and despair. The happiness and playfulness scores were significantly lower than the three negative inner states, while the fearfulness and despair scales did not differ significantly, nor did the playfulness and happiness scales (for *p-*values of the post hoc test see electronic supplementary material, table S6). The growls evoked by a threatening human were also found to be aggressive although the fearfulness ratings did not differ from the aggression in this case. Despair and fearfulness ratings were both medium level, with fearfulness still higher than the playful or happiness scales. The latter two did not differ. Play growls showed the opposite pattern: they were scored equally high on the playful and happy scales, and were given low scores on the aggression, despair and fear scales.

### 3.2. Acoustic differences between the three contexts

In the playful context, there were more individual growls (Poisson GLMM: *F*(2,21) = 17.079; *p* < 0.001) than in food-guarding and threatening contexts. Furthermore, play growls were shorter (lognorm GLMM: *F*(2,21) = 8.331; *p* = 0.002) and separated by shorter intervals (lognorm GLMM: *F*(2,21) = 4.413; *p* = 0.025) than the two agonistic growls.

In the case of the fundamental frequency, we found no significant differences between the contexts (lognorm GLMM: *F*(2,21) = 1.035; *p* = 0.373). By contrast, context had a significant effect on formant dispersion (lognorm GLMM: *F*(2,21) = 67.475; *p* < 0.001), d*F* was the lowest in the play growls, the highest in the food-guarding context and threatening growls were in between (for results of post hoc tests see electronic supplementary material, table S5).

### 3.3. The effect of the acoustic parameters

The linear regression analysis showed that in the case of food-guarding growls, none of the emotional scales was affected by any of the measured acoustic parameters.

Call length had a significant negative effect on the happiness ratings of threatening and play growls: shorter growls were rated to be happier. In the case of threatening growls, call length had a similar effect on playfulness ratings, while it had an opposite effect on two negatively valenced scales: longer growls were rated to be more aggressive and fearful.

IGI had an effect only within play growls. The two positive scales were affected positively (although for playfulness we found only a non-significant trend): growl bouts with longer pauses were rated to be happier. By contrast, we found a negative non-significant trend effect on fearfulness ratings.

Fundamental frequency affected only the fearfulness ratings of threatening growls, showing that higher-pitched growls were considered to be more fearful.

Formant dispersion had a negative effect on fearfulness ratings in the case of threatening and play growls. Growls showing larger body size (i.e. having lower formant dispersion) were considered to be more fearful. In play growls, we found a similar non-significant trend on ratings of aggression, while a significant, but opposite effect on the two positively valenced scales. Growl bouts showing a smaller apparent body size (i.e. having higher formant dispersion) were rated to be more happy and playful (figure 2, for model details see electronic supplementary material, results and table S6).

**Figure:**
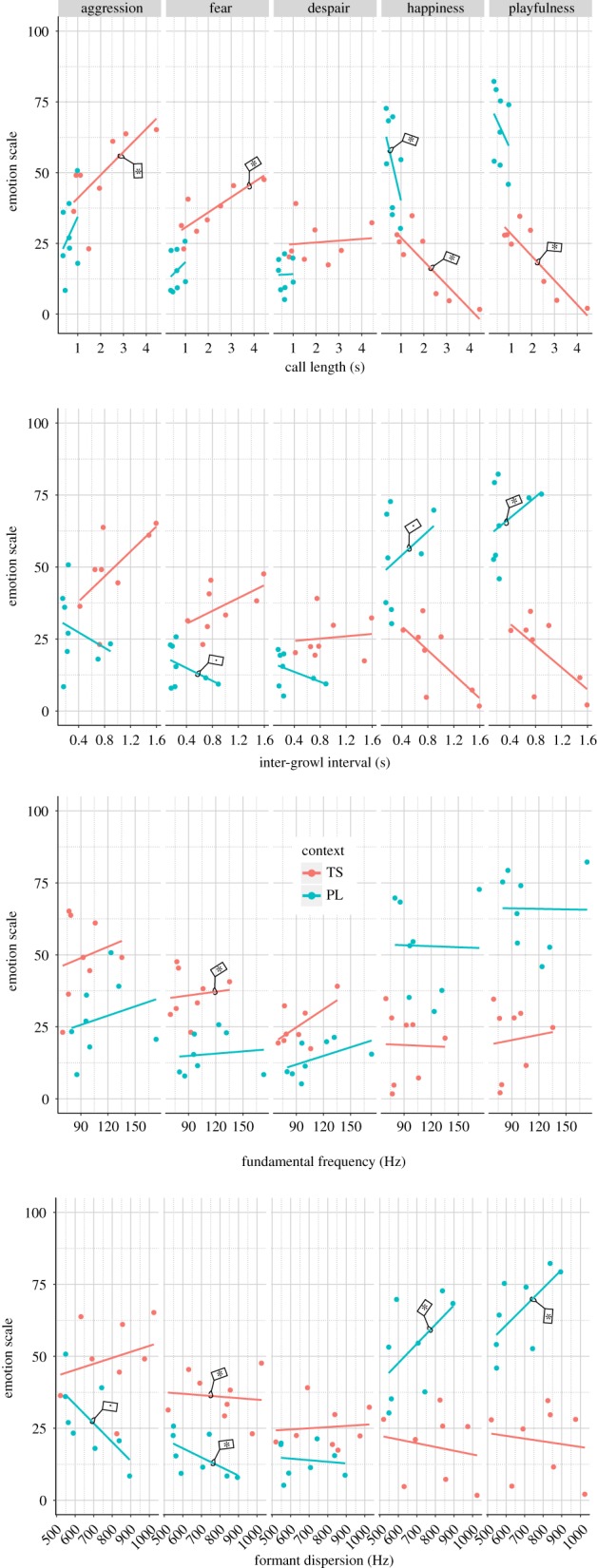
The effect of acoustic parameters on the emotion ratings in threatening and play growls. Significant partial regressions are flagged with an asterisk, non-significant trends are flagged with a dot. Threatening stranger (TS) context indicated in red, play (PL) in blue.

### 3.4. Context recognition

**Figure 3.**
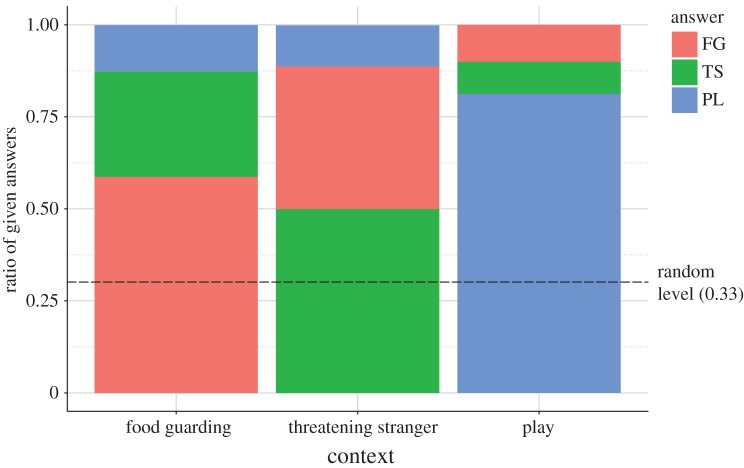
Distribution of context choices of the participants. (FG: food guarding; TS: threatening stranger; PL: play.) The confusion of the two agonistic growls is clearly visible.

Overall, participants classified correctly 63% of the growl samples, which is significantly higher than the 33% chance level (Binomial test: *p* < 0.001). Each of the growl types was also recognized above chance level. Human listeners classified 81% of the play growls correctly, but the food guarding (60%) and the threatening (50%) growls were more difficult to recognize correctly. These results show that participants distinguished play growls more easily, compared with the two agonistic ones and the confusion matrix showed that a relatively high amount of threatening growls were considered to be food guarding and vice versa (figure 3).

Contrary to the case of the emotional ratings, the final GLMM (*F*(4;235) = 6.796; *p* < 0.001) showed significant effects of demographic variables on the context recognition. Three main effects proved to be significant: the context (*F*(2;235) = 8.977; *p* < 0.001), the gender (*F*(1;235) = 6.188; *p* = 0.014) and the dog-ownership status of the participant (*F*(1;235) = 7.765; *p* = 0.006). The post hoc test showed that—as the confusion matrix suggested—the participants were significantly more successfully at recognizing the play context, while there was no difference between the two agonistic contexts. We found that women and dog owners performed better in the recognition task, while dog bite history of the participants had no effect.

## 4. Discussion

The inner-state scaling results reflected that our participants could attribute emotions to growls matching their assumed emotional background, and they were also able to identify the correct social contexts of the growls above chance. They rated the play growls high on the happiness and the playfulness scales. The fear and despair ratings were moderate in both agonistic contexts. Additionally, these agonistic contexts were rated high on aggression, but the food-guarding growls were judged to be the most aggressive. In the threatening growls, fear and aggression scores did not differ from each other. These latter findings are especially interesting in the light of our earlier studies where dogs reacted differently to food-guarding and threatening growls, although we could not detect significant differences between the acoustic parameters (CL, *f*_0_, d*F*, harmonics-to-noise ratio) of the two agonistic contexts [27]. Based on our earlier and present results, we can conclude that both humans and dogs can perceive the difference between the two agonistic contexts based solely on acoustic information. This can be determined by the formant information, as we found in the present sample that threatening growls had significantly lower formant dispersion than food-guarding growls.

Our results also show that the listeners' ratings were mainly affected by the length of the growls and the rhythm of the growl sequence. This finding is in accordance with the results of Taylor *et al*. [33] who showed that humans could categorize and attribute inner state to the resynthesized growl bouts in which the IGIs were similar to natural growl sequences. Nicastro & Owren [16] showed a similar tendency in context recognition of cat vocalizations. In the case of dog barks, rhythm based on inter-bark intervals was found as an important cue for humans to assess the dogs' inner state [34]: longer pauses between individual barks resulted in lower scores of aggression. We found a similar but context-dependent pattern in our growl playbacks. Play growls were usually characterized by fast pulsing growl sequences with short intervals [33], and this was accompanied with short individual growls. As the call length is generally short (around 0.2 s) and less variable among single barks, and their tonality and fundamental frequency can change in a wider range, it is possible that these latter parameters dominate in encoding the emotional load of barks, while growls are more variable in temporal patterns (but less variable in tonality and pitch) and this plays a more important role in emotion assessment. Temporal patterns can also be a factor in the better success rate of context recognition in our study compared with Taylor *et al*.'s. An important difference to their study is that the growl sequences they were using contained growls of uniform length. However, natural growls differ in length; this may provide additional help for humans in deciphering the context. Our recent findings also support this, as in the case of playbacks of various dog vocalizations, the sounds containing shorter calls (along with shorter inter-call intervals) were rated to be more positive [22]. It was found in several other species that besides call length, inter-call intervals can be important indicators of arousal level, because the pauses between utterances shorten with a rise in arousal (mongoose: [38], hyena: [39], baboon: [40] pig: [14]). Nevertheless, studies showing the link between these temporal acoustic variables and emotional valence are scarce thus far.

Within growl types we found slightly different patterns in the effect of acoustic parameters. In food-guarding growls we found no effect of the acoustic structure on the emotion assessment. This could be due to the fact that these highly aggressive, repellent growls (see [27]) are more homogeneous across callers, therein providing more reliable information about the inner state and physical attributes of the individual due to their role in agonistic communication, than the growls used in the other two contexts. In such a competitive and dangerous context, the honest and unambiguous communication of the inner state is favourable and more adaptive, especially considering that these growls are short-range calls, used mostly within visual contact, when any manipulation attempt would be in vain [41]. Alternatively, it is possible that the relatively low number of sound samples used caused low variance, hiding any relationship between the acoustics and the emotion ratings; however, as we could show consistent effects in the other contexts (with the same number of sound samples), this seems to be unlikely. Also the other two contexts possibly have a more uncertain emotional background (threatening: aggression mixed with fear; play: playful aggression) which can be reflected in the acoustic structure of these growls [42], and the listeners seem to be sensitive to this information. This possibility is supported by our recent finding that dogs tend to growl in an acoustically different way towards male and female threatening strangers [43], who may represent different levels of apparent danger to dogs. Present findings show that growl length and the IGI had a significant effect within threatening and play contexts: longer, faster pulsing growls are rated to be more aggressive or fearful in threatening growls and less positively valenced within both contexts. In the threatening stranger context, we found an effect of pitch on the fearfulness scale: listeners rated growls with higher fundamental frequencies to be more fearful. Similarly to this, our earlier findings showed that dogs tend to emit shorter growls when facing a male stranger (and supposedly more threatening) than female strangers [43]. We can assume that the rhythm and the frequency components act in interaction during emotion communication, while the length of the growls provide valence information and the pitch of the growls helps to position the growls on the aggression--fear scale (probably approach–withdrawal scale on the neurological level; see [44]). These findings are in line with results on pig vocalizations that follow similar acoustic patterns (longer, higher-pitched, tonal calls associated with negative contexts) and humans also recognize the context of pig vocalizations based on these acoustic features [13]. Additionally, in goats, a higher level of arousal resulted in higher-pitched vocalizations [45], and our study with playback of various dog vocalizations led to similar results [22]. Thus, we can conclude that these data fit into Morton's theory about the existence of general rules of vocal emotion communication [46].

In our study, emotional ratings were affected somewhat differently by the dogs' size cues (formant dispersion) than that reported by Taylor *et al.* [32]. We found that in both the threatening stranger and playful growls, lower formant dispersion (larger apparent size) was associated with higher level of fear and lower level of playfulness, while the association between higher level of aggression and lower formants was found only in play growls, in contrast with Taylor *et al*.'s finding that threat growls showing a larger apparent size were rated to be more aggressive. This discrepancy may be explained by the fact that Taylor *et al.* utilized resynthesized individual growls for which the formants and fundamental frequency were manipulated in order to indicate larger or smaller individuals. This may have resulted in more clear size cues for the listener, while in our natural growls this information may have been overshadowed by other acoustic features such as the length or pitch variation of the growls. Also, as Taylor *et al.* did not provide a fear scale in their study, we cannot directly compare their results with ours in this case. We earlier found that the apparent body size in play growls is somewhat exaggerated and dogs assess the callers to be larger than their actual size based on this acoustic parameter [42]. We can assume that in such playful contexts the ‘enlarged’ body size in the growls shows enhanced aggression; however, other parameters like pitch and growl length, maintain the playfulness in the interaction. Thus our results indicate that humans perceive the emotional background accordingly.

We also found that humans successfully recognized the context of dog growls, although their performance was lower in the case of the agonistic growl types. Their error pattern clearly showed that most of the wrong choices were intermixes of the food-guarding and threatening stranger situations, which is not surprising as these two agonistic contexts are closer to each other based on their assumed emotional valence and also their acoustic features [27]. Female participants seem to have an advantage in the recognition of the context, in contrast with the lack of gender-effect on the emotion ratings. It is known that women have a higher emotional sensitivity [35,47,48], and probably this higher sensitivity can help to differentiate better the context of the growls, although it would still be worthy to investigate how the emotion assessment of non-human animal vocalizations and context recognition relate to each other.

Additionally we found that, in contrast with the case of dog barks [15], the individual dog-related experience had a positive effect on the performance of the participants. Dog owners recognized better the context of the growls compared with participants who did not own a dog, which is probably due to their extended experiences with dog growls. It is possible that the differentiation of less variable growls needs more cognitive effort than is required for barks, and due to this we can observe this effect only in the former case. Besides this, it is also possible that this is a consequence of the different probabilities of occurrence or accessibility between these two vocalization types. Barks are by far more common vocalizations, as well as being loud, long-distance calls, thus non-owners can even involuntarily obtain experience with them, whereas growls are more ‘intimate’ and less common, and can only be heard in short-range one-to-one interactions—which makes it harder to gain experience without close and repeated contact with dogs. Scheumann *et al.* [19] also found that prior experience with a given animal vocalization affects the recognition abilities of human listeners, moreover Tallet *et al.* [18] found the same in the case of pig vocalizations.

## 5. Conclusion

Our findings emphasize that although emotions may have common acoustic encoding, deciphering of the contextual information of another species' vocal behaviour also involves learning. This phenomenon has already been seen in the case of assessing the emotions in species with different levels of familiarity to human listeners, but now we also found evidence for it in less common types of dog vocalizations. Our results may also indicate that dogs communicate honestly their size and inner state in serious contest situations, where confrontation would be costly, such as during guarding of their food from another dog. At the same time, in contexts with assumedly more uncertain inner states, such as in play or when threatened by a stranger, they may manipulate certain key parameters in their growls for an exaggerated aggressive and playful expression. According to our results, adult humans seem to understand and respond accordingly to this acoustic information during cross-species interactions with dogs.

## Supplementary Material

Supplementary Results

## Supplementary Material

Supplementary Tables

## Supplementary Material

Supplementary data

## Data accessibility

All data are available in the electronic supplementary material.

## References

[1] Briefer EF 2012 Vocal expression of emotions in mammals: mechanisms of production and evidence. J. Zool. 288, 1–20. (doi:10.1111/j.1469-7998.2012.00920.x)

[2] Taylor AM, Reby D 2010 The contribution of source-filter theory to mammal vocal communication research. J. Zool. 280, 221–236. (doi:10.1111/j.1469-7998.2009.00661.x)

[3] Fitch WT, Hauser MD 2003 Unpacking ‘honesty’vertebrate vocal production and the evolution of acoustic signals. In Acoustic communication (eds Simmons AM, Popper AN, Fay RR), pp. 65–137. New York, NY: Springer.

[4] Fecteau S, Belin P, Joanette Y, Armony JL 2007 Amygdala responses to nonlinguistic emotional vocalizations. Neuroimage 36, 480–487. (doi:10.1016/j.neuroimage.2007.02.043) 17442593 10.1016/j.neuroimage.2007.02.043

[5] Schirmer A, Kotz SA 2006 Beyond the right hemisphere: brain mechanisms mediating vocal emotional processing. Trends Cogn. Sci. 10, 24–30. (doi:10.1016/j.tics.2005.11.009) 16321562 10.1016/j.tics.2005.11.009

[6] Belin P, Fecteau S, Charest I, Nicastro N, Hauser MD, Armony JL 2008 Human cerebral response to animal affective vocalizations. Proc. R. Soc. B 275, 473–481. (doi:10.1098/rspb.2007.1460) 10.1098/rspb.2007.1460 PMC2596811 18077254

[7] Andics A, Gácsi M, Faragó T, Kis A, Miklósi Á 2014 Voice-sensitive regions in the dog and human brain are revealed by comparative FMRI. Curr. Biol. 24, 574–578. (doi:10.1016/j.cub.2014.01.058) 24560578 10.1016/j.cub.2014.01.058

[8] Shriner WM 1998 Yellow-bellied marmot and golden-mantled ground squirrel responses to heterospecific alarm calls. Anim. Behav. 55, 529–536. (doi:10.1006/anbe.1997.0623) 9514669 10.1006/anbe.1997.0623

[9] Müller CA, Manser MB 2008 The information banded mongooses extract from heterospecific alarms. Anim. Behav. 75, 897–904. (doi:10.1016/j.anbehav.2007.07.012)

[10] Fichtel C 2004 Reciprocal recognition of sifaka (*Propithecus verreauxi verreauxi*) and redfronted lemur (*Eulemur fulvus rufus*) alarm calls. Anim. Cogn. 7, 45–52. (doi:10.1007/s10071-003-0180-0) 12827548 10.1007/s10071-003-0180-0

[11] Lingle S, Riede T 2014 Deer mothers are sensitive to infant distress vocalizations of diverse mammalian species. Am. Nat. 184, 510–522. (doi:10.1086/677677) 25226186 10.1086/677677

[12] Linnankoski I, Laakso M-L, Aulanko R, Leinonen L 1994 Recognition of emotions in macaque vocalizations by children and adults. Lang. Commun. 14, 183–192. (doi:10.1016/0271-5309(94)90012-4)

[13] Tallet C, Linhart P, Policht R, Hammerschmidt K, Šimeček P, Kratinova P, Špinka M 2013 Encoding of situations in the vocal repertoire of piglets (*Sus scrofa*): a comparison of discrete and graded classifications. PLoS ONE 8, e71841 (doi:10.1371/journal.pone.0071841) 23967251 10.1371/journal.pone.0071841 PMC3742501

[14] Maruščáková IL, Linhart P, Ratcliffe VF, Tallet C, Reby D, Špinka M 2015 Humans (*Homo sapiens*) judge the emotional content of piglet (*Sus scrofa domestica*) calls based on simple acoustic parameters, not personality, empathy, nor attitude toward animals. J. Comp. Psychol. 129, 121–131. (doi:10.1037/a0038870) 25798794 10.1037/a0038870

[15] Pongrácz P, Molnár C, Miklósi Á, Csányi V 2005 Human listeners are able to classify dog (*Canis familiaris*) barks recorded in different situations. J. Comp. Psychol. 119, 136–144. (doi:10.1037/0735-7036.119.2.136) 15982157 10.1037/0735-7036.119.2.136

[16] Nicastro N, Owren MJ 2003 Classification of domestic cat (*Felis catus*) vocalizations by naive and experienced human listeners. J. Comp. Psychol. 117, 44–52. (doi:10.1037/0735-7036.117.1.44) 12735363 10.1037/0735-7036.117.1.44

[17] Ellis SLH, Swindell V, Burman OHP 2015 Human classification of context-related vocalizations emitted by familiar and unfamiliar domestic cats: an exploratory study. Anthrozoos 28, 625–634. (doi:10.1080/08927936.2015.1070005)

[18] Tallet C, Špinka M, Maruščáková IL, Šimeček P 2010 Human perception of vocalizations of domestic piglets and modulation by experience with domestic pigs (*Sus scrofa*). J. Comp. Psychol. 124, 81–91. (doi:10.1037/a0017354) 20175599 10.1037/a0017354

[19] Scheumann M, Hasting AS, Kotz SA, Zimmermann E 2014 The voice of emotion across species: how do human listeners recognize animals’ affective states? PLoS ONE 9, e91192 (doi:10.1371/journal.pone.0091192) 24621604 10.1371/journal.pone.0091192 PMC3951321

[20] Pongrácz P, Molnár C, Dóka A, Miklósi Á 2011 Do children understand man's best friend? Classification of dog barks by pre-adolescents and adults. Appl. Anim. Behav. Sci. 135, 95–102. (doi:10.1016/j.applanim.2011.09.005)

[21] Molnár C, Pongrácz P, Miklósi Á 2009 Seeing with ears: sightless humans’ perception of dog bark provides a test for structural rules in vocal communication. Q. J. Exp. Psychol. 63, 1004–1013. (doi:10.1080/17470210903168243) 10.1080/17470210903168243 19760535

[22] Faragó T, Andics A, Devecseri V, Kis A, Gácsi M, Miklósi Á 2014 Humans rely on the same rules to assess emotional valence and intensity in conspecific and dog vocalizations. Biol. Lett. 10, 20130926 (doi:10.1098/rsbl.2013.0926) 24402716 10.1098/rsbl.2013.0926 PMC3917336

[23] Wang G-D et al. 2016 Out of southern East Asia: the natural history of domestic dogs across the world. Cell Res. 26, 21–33. (doi:10.1038/cr.2015.147) 26667385 10.1038/cr.2015.147 PMC4816135

[24] Pongrácz P, Molnár C, Miklósi Á 2010 Barking in family dogs: an ethological approach. Vet. J. 183, 141–147. (doi:10.1016/j.tvjl.2008.12.010) 19181546 10.1016/j.tvjl.2008.12.010

[25] Cohen JA, Fox MW 1976 Vocalizations in wild canids and possible effects of domestication. Behav. Process. 1, 77–92. (doi:10.1016/0376-6357(76)90008-5) 10.1016/0376-6357(76)90008-5 24923546

[26] Faragó T, Townsend SW, Range F 2014 The information content of wolf (and dog) social communication. In Biocommunication of animals (ed. Witzany G), pp. 41–62. Dordrecht, The Netherlands: Springer (doi:10.1007/978-94-007-7414-8_4)

[27] Faragó T, Pongrácz P, Range F, Virányi Z, Miklósi Á 2010 ‘The bone is mine’: affective and referential aspects of dog growls. Anim. Behav. 79, 917–925. (doi:10.1016/j.anbehav.2010.01.005)

[28] Faragó T, Pongrácz P, Miklósi Á, Huber L, Virányi Z, Range F 2010 Dogs’ expectation about signalers’ body size by virtue of their growls. PLoS ONE 5, e15175 (doi:10.1371/journal.pone.0015175) 21179521 10.1371/journal.pone.0015175 PMC3002277

[29] Taylor AM, Reby D, McComb K 2011 Cross modal perception of body size in domestic dogs (*Canis familiaris*). PLoS ONE 6, e17069 (doi:10.1371/journal.pone.0017069) 21359228 10.1371/journal.pone.0017069 PMC3040207

[30] Taylor AM, Reby D, McComb K 2010 Size communication in domestic dog, *Canis familiaris*, growls. Anim. Behav. 79, 205–210. (doi:10.1016/j.anbehav.2009.10.030)

[31] Taylor AM, Reby D, McComb K 2008 Human listeners attend to size information in domestic dog growls. J. Acoust. Soc. Am. 123, 2903–2909. (doi:10.1121/1.2896962) 18529206 10.1121/1.2896962

[32] Taylor AM, Reby D, McComb K 2010 Why do large dogs sound more aggressive to human listeners: acoustic bases of motivational misattributions. Ethology 116, 1155–1162. (doi:10.1111/j.1439-0310.2010.01829.x)

[33] Taylor AM, Reby D, McComb K 2009 Context-related variation in the vocal growling behaviour of the domestic dog (*Canis familiaris*). Ethology 115, 905–915. (doi:10.1111/j.1439-0310.2009.01681.x)

[34] Pongrácz P, Molnár C, Miklósi Á 2006 Acoustic parameters of dog barks carry emotional information for humans. Appl. Anim. Behav. Sci. 100, 228–240. (doi:10.1016/j.applanim.2005.12.004)

[35] Lithari C, Frantzidis CA, Papadelis C, Vivas AB, Klados MA, Kourtidou-Papadeli C, Pappas C, Ioannides AA, Bamidis PD 2010 Are females more responsive to emotional stimuli? A neurophysiological study across arousal and valence dimensions. Brain Topogr. 23, 27–40. (doi:10.1007/s10548-009-0130-5) 20043199 10.1007/s10548-009-0130-5 PMC2816804

[36] Boersma P, Weenink D 2014 Praat: doing phonetics by computer. See www.praat.org.

[37] Maxwell C 1978 Sensitivity and accuracy of the visual analogue scale: a psycho-physical classroom experiment. Br. J. Clin. Pharmacol. 6, 15–24. (doi:10.1111/j.1365-2125.1978.tb01676.x) 666944 10.1111/j.1365-2125.1978.tb01676.x PMC1429397

[38] Manser MB 2001 The acoustic structure of suricates’ alarm calls varies with predator type and the level of response urgency. Proc. R. Soc. Lond. B 268, 2315–2324. (doi:10.1098/rspb.2001.1773) 10.1098/rspb.2001.1773 PMC1088882 11703871

[39] Theis KR, Greene KM, Benson-Amram SR, Holekamp KE 2007 Sources of variation in the long-distance vocalizations of spotted hyenas. Behaviour 144, 557–584. (doi:10.1163/156853907780713046)

[40] Meise K, Keller C, Cowlishaw G, Fischer J 2011 Sources of acoustic variation: implications for production specificity and call categorization in chacma baboon (*Papio ursinus*) grunts. J. Acoust. Soc. Am. 129, 1631–1641. (doi:10.1121/1.3531944) 21428526 10.1121/1.3531944

[41] Számadó S 2008 How threat displays work: species-specific fighting techniques, weaponry and proximity risk. Anim. Behav. 76, 1455–1463. (doi:10.1016/j.anbehav.2008.07.010)

[42] Bálint A, Faragó T, Dóka A, Miklósi Á, Pongrácz P 2013 ‘Beware, I am big and non-dangerous!’— Playfully growling dogs are perceived larger than their actual size by their canine audience. Appl. Anim. Behav. Sci. 148, 128–137. (doi:10.1016/j.applanim.2013.07.013)

[43] Bálint A, Faragó T, Miklósi Á, Pongrácz P 2016 Threat-level-dependent manipulation of signaled body size: dog growls’ indexical cues depend on the different levels of potential danger. Anim. Cogn. 19, 1115–1131. (doi:10.1007/s10071-016-1019-9) 27473205 10.1007/s10071-016-1019-9

[44] Ehret G, Kurt S 2010 Selective perception and recognition of vocal signals. In Handbook of mammalian vocalization an integrative neuroscience approach (ed. Brudzynski SM), pp. 125–134. London, UK: Academic Press (doi:10.1016/B978-0-12-374593-4.00013-9)

[45] Briefer EF, Tettamanti F, McElligott AG 2015 Emotions in goats: mapping physiological, behavioural and vocal profiles. Anim. Behav. 99, 131–143. (doi:10.1016/j.anbehav.2014.11.002)

[46] Zimmermann E, Leliveld LMC, Schehka S 2013 Toward the evolutionary roots of affective prosody in human acoustic communication: a comparative approach to mammalian voices. In Evolution of emotional communication: from sounds in nonhuman mammals to speech and music in man. (eds Altenmüller E, Schmidt S, Zimmermann E), pp. 116–132. Oxford, UK: Oxford University Press.

[47] Collignon O, Girard S, Gosselin F, Saint-Amour D, Lepore F, Lassonde M 2010 Women process multisensory emotion expressions more efficiently than men. Neuropsychologia 48, 220–225. (doi:10.1016/j.neuropsychologia.2009.09.007) 19761782 10.1016/j.neuropsychologia.2009.09.007

[48] Hampson E, van Anders SM, Mullin LI 2006 A female advantage in the recognition of emotional facial expressions: test of an evolutionary hypothesis. Evol. Hum. Behav. 27, 401–416. (doi:10.1016/j.evolhumbehav.2006.05.002)

