# Supplementary material for: Dog growls express various contextual and affective content for human listeners
Source: R Soc Open Sci. 2017 May 17;4(5):170134. doi: 10.1098/rsos.170134 (PMC5451822; doi:10.1098/rsos.170134)
Supplement: Supplementary Results [file rsos170134supp1.docx]

### Supplementary Results

Table S4 The average (+/-SD) values of acoustic parameters and the emotional ratings within contexts.

|  | food guarding | **threatening** | **play** |
| --- | --- | --- | --- |
| CN | 4.5+/-1.3 | 4.5+/-1.8 | 12.4+/-5.1 |
| CL (s) | 1.8+/-0.7 | 2.1+/-1.3 | 0.6+/-0.3 |
| IGI (s) | 0.9+/-0.3 | 0.9+/-0.4 | 0.4+/-0.3 |
| *f*_0_ (Hz) | 104.6+/-29.9 | 92+/-21.6 | 110.7+/-30.8 |
| d*F* (Hz) | 902.6+/-230.4 | 795+/-170.7 | 678.8+/-137.2 |
| aggression | 63,5+/-32,7 | 48,6+/-31,1 | 29+/-28,9 |
| fear | 36,9+/-31,2 | 37,5+/-30 | 14,9+/-19,2 |
| despair | 28,3+/-28 | 27,2+/-27,8 | 14,1+/-19,9 |
| playfulness | 14,6+/-22,5 | 19,4+/-26,1 | 64,7+/-33,1 |
| happiness | 11,6+/-18,7 | 17,7+/-25,7 | 50,9+/-35,4 |

Table S5. The acoustic differences between the three contexts. The table shows the post-hoc pairwise comparisons (sequential Sidak test) of the contexts. Cells with white background contain significant contrasts. Adjusted p values contrast estimates with 95% confidence interval are reported.

|  | | **threatening** | **play** |
| --- | --- | --- | --- |
| CN | food guarding | p=0.987  0.02 [-2.31;2.35] | p<0.001  -7.80 [-11.24; -4.37] |
|  | threatening |  | p<0.001  -7.82 [-11.26; -4.38] |
| CL | food guarding | p=0.340  -0.22 [-0.69; 0.25] | p=0.001  1.05 [-0.47; 1.62] |
|  | threatening |  | p<0.001  1.27 [0.64; 1.90] |
| IGI | food guarding | p=0.766  -0.05 [-0.39; 0.29] | p=0.004  0.53 [0.19; 0.87] |
|  | threatening |  | p=0.002  0.58 [0.25; 0.92] |
| *f*_0_ | food guarding | p=0.377  10.92 [-14.26; 36.10] | p=0.534  -8.44 [-36.18; 19.31] |
|  | threatening |  | p=0.166  -19.35 [-47.43; 8.72] |
| d*F* | food guarding | p<0.001  175.92 [105.29; 246.54] | p<0.001  399.81 [312.32; 487.28] |
|  | threatening |  | p<0.001  223.89 [140.43; 307.35] |

### Response consistency

We analysed the consistency of the participants' responses with Spearman rank correlation tests for each emotional scale and the recognition success. We found that in each inner state scoring except fearfulness the first and second rating of the same sound sample correlated positively (Spearman correlation tests – playfulness: δ=0.63; p<0.001; fear: δ=.167; p=0.303; aggressiveness: δ=0.59; p<0.001; happiness: δ=0.62; p<0.001; desperate: δ=0.38; p=0.015).

In case of the context questionnaire, again we found that the participants' choices correlated positively between the first and second repetition of the growl samples (Spearman: δ=0.612, p<0.001) suggesting that they mostly chose the same context for both playbacks. The distribution of the first and second guesses were not random (Chi square test: χ^2^(4)=20.658; p<0.001), the food guarding and play contexts dominated the answers, and those growls that were marked as play growls for the first listening, was mostly marked as play growl again (13 from 14). In contrast food guarding was changed to threatening significantly more than to play during the second listening (8 times vs. 3 times from 19). However, their choices were only roughly consistent with the original context of the growls (Figure S1), mainly because the discrimination of the two agonistic growl types was uncertain. Interestingly, the second choices seem to be more accurate: while only 19 subjects classified the growls correctly after the first hearing, for the second round 26 subjects chose the correct context.


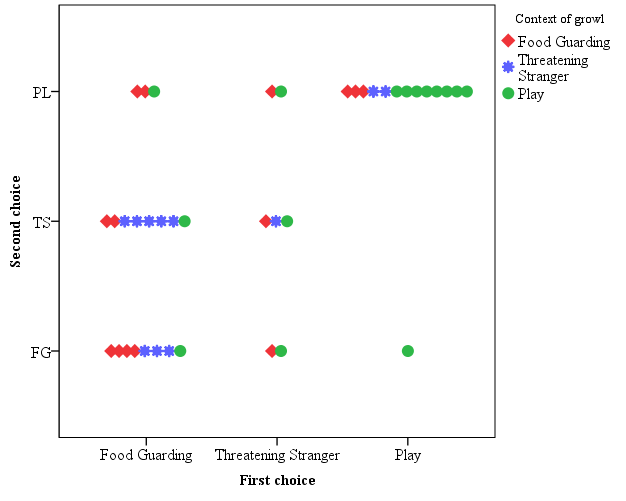


Figure S1. The consistency of the context choices between the first (horizontal axis) and seventh (vertical axis) playback. Each dot represents one participant’s response given on the repeated playback, while the colouring and shape of the dot shows the original context of the growl.

Table S6 The results of the within emotion scale post-hoc tests (Sequential Sidak test). Cells with white background contain significant contrasts. Adjusted p values contrast estimates with 95% confidence interval are reported.

|  | | **threatening** | **play** |
| --- | --- | --- | --- |
| Aggression | food guarding | p=0.022  1.38 [0.20;2.56] | p<0.001  3.97 [2.54;5.41] |
|  | threatening |  | p<0.001  2.59 [1.25;3.93] |
| Fear | food guarding | p=0.176  -0.21 [-1.39;0.96] | p<0.001  2.93 [1.59;4.27] |
|  | threatening |  | p<0.001  3.15 [1.71;4.58] |
| Despair | food guarding | p=0.112  0.08 [-1.10;1.25] | p<0.001  2.12 [0.69;3.56] |
|  | threatening |  | p<0.001  2.05 [0.70;3.39] |
| Playfulness | food guarding | p=0.898  -0.81 [-1.99;0.36] | p=0.001  -6.39 [-7.83;-4.96] |
|  | threatening |  | p<0.001  -5.58 [-6.92;-4.24] |
| Happiness | food guarding | p=0.721  -0.95 [-2.13;0.22] | p<0.001  -5.52 [-6.96;-4.09] |
|  | threatening |  | p<0.001  -4.57 [-5.91;-3.23] |

Table S7 The results of the within context post-hoc tests (Sequential Sidak test). Cells with white background contain significant contrasts. Adjusted p values contrast estimates with 95% confidence interval are reported.

|  | | fear | despair | playfulness | happiness |
| --- | --- | --- | --- | --- | --- |
| food guarding | aggression | p<0.001  2.83 [1.29;4.37] | p<0.001  3.99 [2.35;5.63] | p<0.001  6.21 [4.55;7.87] | p<0.001  6.66 [4.98;8.34] |
|  | fear |  | p=0.103  1.16 [-0.18;2.50] | p<0.001  3.38 [1.80;4.96] | p<0.001  3.83 [2.22;5.44] |
|  | despair |  |  | p=0.001  2.22 [0.79;3.66] | p<0.001  2.67 [1.17;4.17] |
|  | playfulness |  |  |  | 0.453  0.45 [-0.73;1.63] |
| **threatening** | aggression | p=0.078  1.23 [-0.11;2.58] | p<0.001  2.69 [1.10;4.27] | p<0.001  4.02 [2.36;5.68] | p<0.001  4.33 [2.64;6.01] |
|  | fear |  | p=0.061  1.45 [-0.05;2.95] | p<0.001  2.78 [1.17;4.40] | p<0.001  3.09 [1.45;4.73] |
|  | despair |  |  | p=0.077  1.33 [-0.10;2.77] | p=0.031  1.64 [0.10;3.18] |
|  | playfulness |  |  |  | 0.608  0.31 [-0.87;1.48] |
| play | aggression | p=0.009  1.79 [0.36;3.22] | p=0.002  2.14 [0.65;3.64] | p<0.001  -4.15 [-5.73;-2.57] | p<0.001  -2.83 [-4.38;-1.29] |
|  | fear |  | p=0.555  0.35 [-0.82;1.53] | p<0.001  -5.94 [-7.60;-4.28] | p<0.001  -4.62 [-6.24;-3.01] |
|  | despair |  |  | p<0.001  -6.30 [-7.98;-4.61] | p<0.001  -4.98 [-6.62;-3.34] |
|  | playfulness |  |  |  | p=0.055  1.32 [-0.02;2.66] |

Table S8 The results of Linear Regression models showing the effect of acoustic variables on emotional ratings. The results of the final model after backwards elimination are shown. Results within food guarding context, and despair scale are omitted, because here the null-models were the final ones after model selection. Significant partial effects are shown in italics.

|  | | **threatening** | | | **play** | | |
| --- | --- | --- | --- | --- | --- | --- | --- |
| **aggression** | **Model** | **R^2^** | **F** | **p** | **R^2^** | **F** | **p** |
|  |  | ,503 | 6,082 | ,049 | ,426 | 4,452 | ,079 |
|  | **Partials** | **Std. β** | **t** | **p** | **Std. β** | **t** | **p** |
|  | **CL** | *,710* | *2,466* | *,049* |  |  |  |
|  | **IGI** |  |  |  |  |  |  |
|  | ***f*_0_** |  |  |  |  |  |  |
|  | **d*F*** |  |  |  | -,653 | -2,110 | ,079 |
| **fear** | **Model** | **R^2^** | **F** | **p** | **R^2^** | **F** | **p** |
|  |  | ,959 | 31,316 | ,003 | ,675 | 5,204 | ,060 |
|  | **Partials** | **Std. β** | **t** | **p** | **Std. β** | **t** | **p** |
|  | **CL** | *1,115* | *9,625* | *,001* |  |  |  |
|  | **IGI** |  |  |  | -,593 | -2,269 | ,073 |
|  | ***f*_0_** | *,398* | *3,763* | *,020* |  |  |  |
|  | **d*F*** | *-,502* | *-4,513* | *,011* | *-,719* | *-2,747* | *,040* |
| **playfulness** | **Model** | **R^2^** | **F** | **p** | **R^2^** | **F** | **p** |
|  |  | ,765 | 19,497 | ,004 | ,693 | 5,652 | ,052 |
|  | **Partials** | **Std. β** | **t** | **p** | **Std. β** | **t** | **p** |
|  | **CL** | *-,874* | *-4,416* | *,004* |  |  |  |
|  | **IGI** |  |  |  | ,554 | 2,178 | ,081 |
|  | ***f*_0_** |  |  |  |  |  |  |
|  | **d*F*** |  |  |  | *,760* | *2,988* | *,031* |
| **happiness** | **Model** | **R^2^** | **F** | **p** | **R^2^** | **F** | **p** |
|  |  | ,723 | 15,666 | ,007 | ,889 | 10,688 | ,022 |
|  | **Partials** | **Std. β** | **t** | **p** | **Std. β** | **t** | **p** |
|  | **CL** | *-,850* | *-3,958* | *,007* | *-,665* | *-3,464* | *,026* |
|  | **IGI** |  |  |  | *,751* | *4,064* | *,015* |
|  | ***f*_0_** |  |  |  |  |  |  |
|  | **d*F*** |  |  |  | *,497* | *2,784* | *,050* |
