# Supplementary material for: Dog growls express various contextual and affective content for human listeners
Source: R Soc Open Sci. 2017 May 17;4(5):170134. doi: 10.1098/rsos.170134 (PMC5451822; doi:10.1098/rsos.170134)
Supplement: Supplementary Tables [file rsos170134supp2.docx]

**Supplementary tables**

Table S1 The personal and dog experience data of the participants.

| **ID** | **gender** | **age** | **dog experience** | **bite history** |
| --- | --- | --- | --- | --- |
| 1 | 2 | 23 | 1 | 0 |
| 2 | 2 | 28 | 1 | 0 |
| 3 | 2 | 28 | 1 | 0 |
| 4 | 2 | 23 | 0 | 0 |
| 5 | 1 | 21 | 1 | 1 |
| 6 | 2 | 33 | 1 | 1 |
| 7 | 2 | 21 | 1 | 1 |
| 8 | 2 | 24 | 1 | 0 |
| 9 | 1 | 54 | 1 | 0 |
| 10 | 2 | 53 | 1 | 1 |
| 11 | 2 | 23 | 1 | 1 |
| 12 | 2 | 20 | 0 | 1 |
| 13 | 2 | 19 | 0 | 0 |
| 14 | 1 | 20 | 1 | 0 |
| 15 | 2 | 29 | 1 | 1 |
| 16 | 1 | 26 | 1 | 1 |
| 17 | 2 | 26 | 0 | 0 |
| 18 | 1 | 26 | 1 | 0 |
| 19 | 2 | 22 | 1 | 1 |
| 20 | 1 | 21 | 1 | 0 |
| 21 | 1 | 26 | 1 | 1 |
| 22 | 2 | 26 | 1 | 1 |
| 23 | 1 | 28 | 1 | 0 |
| 24 | 1 | 29 | 1 | 0 |
| 25 | 1 | 26 | 1 | 1 |
| 26 | 1 | 26 | 0 | 0 |
| 27 | 1 | 27 | 0 | 0 |
| 28 | 1 | 22 | 1 | 0 |
| 29 | 2 | 22 | 1 | 0 |
| 30 | 2 | 27 | 1 | 0 |
| 31 | 2 | 24 | 1 | 1 |
| 32 | 2 | 23 | 0 | 0 |
| 33 | 1 | 24 | 0 | 0 |
| 34 | 2 | 21 | 0 | 0 |
| 35 | 2 | 30 | 1 | 1 |
| 36 | 2 | 20 | 1 | 0 |
| 37 | 2 | 22 | 1 | 0 |
| 38 | 2 | 23 | 1 | 1 |
| 39 | 2 | 37 | 1 | 0 |
| 40 | 2 | 20 | 1 | 1 |

Table S2 The acoustic paramteres of the growl samples used in the playbacks (ID – dog, CN – number of growls in the bout, CL – average length of the growls, IGI – average inter-growl-intervals in the bouts, *f*_0_ – average fundamental frequency, d*F* average formant dispersion)

| **Growl ID** | **Context** | **ID** | **sex** | **age (years)** | **weight (kg)** | **height (cm)** | **CN** | **CL** | **IGI** | *f*_0_ | d*F* |
| --- | --- | --- | --- | --- | --- | --- | --- | --- | --- | --- | --- |
| a1 | food guarding | 1 | 2 | 7 | 13 | 44 | 7 | 0.73 | 0.91 | 125.5 | 600.1 |
| a2 |  | 2 | 1 | 10 | 27 | 63 | 6 | 1.25 | 0.62 | 76.0 | 1207.8 |
| a3 |  | 3 | 2 | 7 | 17 | 50 | 4 | 1.55 | 1.31 | 94.5 | 934.9 |
| a4 |  | 4 | 1 | 2.5 | 29 | 64 | 4 | 1.96 | 1.20 | 158.0 | 1236.0 |
| a5 |  | 5 | 1 | 5.5 | 32 | 61 | 4 | 1.71 | 0.80 | 90.6 | 853.1 |
| a6 |  | 6 | 2 | 5 | 34 | 64 | 4 | 2.27 | 0.54 | 66.3 | 859.9 |
| a7 |  | 7 | 1 | 2.5 | 30 | 60 | 4 | 2.05 | 0.86 | 101.5 | 637.3 |
| a8 |  | 8 | 2 | 4.5 | 12 | 45 | 3 | 3.23 | 0.54 | 124.6 | 891.8 |
| b1 | threatening | 1 | 2 | 7 | 13 | 44 | 7 | 0.82 | 0.42 | 76.4 | 520.4 |
| b2 |  | 9 | 2 | 2.5 | 15 | 46 | 2 | 4.44 | 1.59 | 77.0 | 1024.0 |
| b3 |  | 10 | 1 | 5 | 20 | 54 | 4 | 1.95 | 1.00 | 99.8 | 838.3 |
| b4 |  | 3 | 2 | 7 | 17 | 50 | 5 | 1.49 | 0.72 | 69.9 | 824.9 |
| b5 |  | 11 | 2 | 1 | 10 | 46 | 3 | 2.54 | 1.48 | 105.9 | 855.4 |
| b6 |  | 5 | 1 | 5.5 | 32 | 61 | 6 | 1.11 | 0.75 | 135.3 | 691.2 |
| b7 |  | 6 | 2 | 5 | 34 | 64 | 3 | 3.11 | 0.78 | 78.9 | 631.2 |
| b8 |  | 12 | 1 | 4 | 10 | 35 | 6 | 0.92 | 0.65 | 92.5 | 974.2 |
| c1 | play | 13 | 1 | 2.5 | 19 | 56 | 9 | 0.98 | 0.24 | 123.4 | 549.0 |
| c2 |  | 2 | 1 | 10 | 27 | 63 | 6 | 1.00 | 0.70 | 99.9 | 707.2 |
| c3 |  | 14 | 2 | 2 | 18 | 48 | 20 | 0.35 | 0.22 | 173.1 | 837.4 |
| c4 |  | 15 | 2 | 3 | 17 | 43 | 6 | 0.64 | 0.89 | 79.9 | 590.3 |
| c5 |  | 16 | 2 |  | 19 | 59 | 16 | 0.45 | 0.17 | 85.7 | 893.8 |
| c6 |  | 3 | 2 | 7 | 17 | 50 | 12 | 0.62 | 0.24 | 95.7 | 562.6 |
| c7 |  | 17 | 1 | 4 | 35 | 60 | 16 | 0.36 | 0.18 | 96.3 | 547.7 |
| c8 |  | 18 | 2 | 3 | 25 | 55 | 14 | 0.62 | 0.15 | 131.9 | 742.7 |

Table S3 The order of growl samples in the 20 playback sessions. The cells refer to the growl sample ID in Table S2.

| **Session** | **Order of growls** | | | | | | |
| --- | --- | --- | --- | --- | --- | --- | --- |
|  | **1** | **2** | **3** | **4** | **5** | **6** | ***7*** |
| **1** | b6 | c8 | c7 | a5 | b2 | a4 | *b6* |
| **2** | c1 | a6 | b6 | c6 | b7 | a4 | *c1* |
| **3** | a5 | c7 | c3 | b4 | b8 | a6 | *a5* |
| **4** | c5 | b4 | c3 | a7 | a8 | b3 | *c5* |
| **5** | a6 | c5 | b8 | b6 | a1 | c1 | *a6* |
| **6** | b5 | c2 | c6 | a5 | a4 | b4 | *b5* |
| **7** | c4 | a7 | c2 | a6 | b4 | b7 | *c4* |
| **8** | a4 | c8 | c4 | b6 | a5 | b7 | *a4* |
| **9** | a8 | c1 | a6 | c2 | b6 | b4 | *a8* |
| **10** | a2 | c3 | a5 | b7 | c8 | b5 | *a2* |
| **11** | a7 | c5 | c1 | a3 | b2 | b1 | *a7* |
| **12** | c8 | b3 | c4 | b1 | a7 | a1 | *c8* |
| **13** | b7 | a8 | b2 | a1 | c7 | c5 | *b7* |
| **14** | c3 | c8 | a7 | a1 | b2 | b5 | *c3* |
| **15** | b1 | b8 | c2 | c6 | a1 | a2 | *b1* |
| **16** | a3 | c1 | c6 | b5 | b2 | a2 | *a3* |
| **17** | c7 | c5 | b1 | b3 | a8 | a3 | *c7* |
| **18** | b8 | a2 | b5 | a8 | c4 | c7 | *b8* |
| **19** | b3 | c6 | b1 | a2 | c4 | a3 | *b3* |
| **20** | c2 | a3 | b8 | a4 | c3 | b3 | *c2* |
